# Supplementary figures and images for: Properties of Individual Hippocampal Synapses Influencing NMDA-Receptor Activation by Spontaneous Neurotransmission
Source: eNeuro. 2019 May 29;6(3):ENEURO.0419-18.2019. doi: 10.1523/ENEURO.0419-18.2019 (PMC6541874; doi:10.1523/ENEURO.0419-18.2019)

Extended Data Figure 1-1

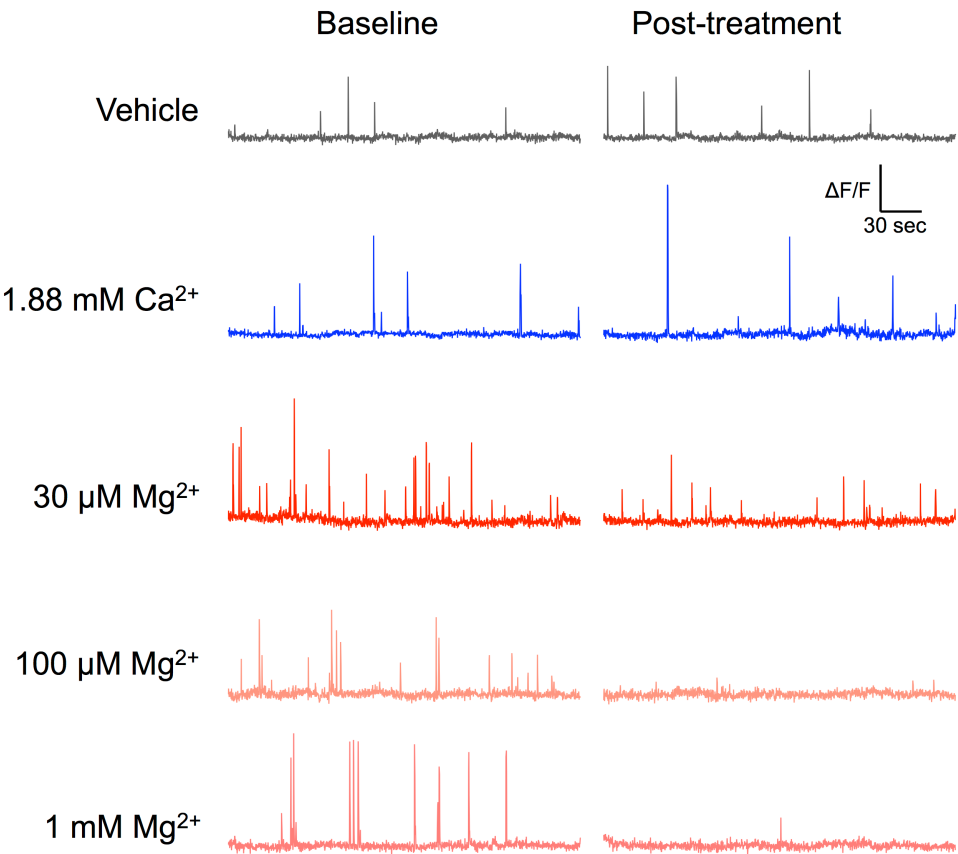

Supplement: Figure 1-1 — Example mSCaT traces from cells in Figure 1. Example mSCaT traces for data from Figure 1I. Download Figure 1-1, PDF file. [file sup_enu-eN-NWR-0419-18-s01.pdf]
